# Investigation of γ-Aminopropyltriethoxysilane (APTES)-Modified Halloysite Nanotubes on the Reinforcement of Halloysite/Polypropylene (PP) Nanocomposites

**DOI:** 10.3390/polym16233332

**Published:** 2024-11-27

**Authors:** Zuguo Bao, Yunxiang Yan, Weijian Han

**Affiliations:** 1 Key Laboratory for Light-Weight Materials, Nanjing Tech University, Nanjing 210009, China; 2 Yangtze Delta Region Institute of Advanced Materials, Suzhou 215133, China

**Keywords:** halloysite, APTES, rheology, crystallization, mechanical properties

## Abstract

Halloysite was successfully grafted with silane APTES using both wet and dry modification methods. Among them, wet modification using aqueous solution possessed the highest modification efficiency and grafting ratio. Morphological observations demonstrated that APTES grafting on halloysite improved both the filler dispersion and interfacial compatibility in polymer composites. The rheology and crystallization behavior indicated that silane modification improved the halloysite dispersion in the polypropylene (PP) matrix and enhanced the interfacial bonding. Furthermore, the modified halloysite also improved the thermal stability of the PP composites, showing elevated decomposition temperatures of PP. The mechanical properties of halloysite/PP nanocomposites were apparently improved with APTES modification. The filler content of the nanocomposites was finally optimized to 2% by considering trade-offs of the tensile, flexural, and Izod impact properties.

## 1. Introduction

Polypropylene (PP) is the most widely used commercial plastic in the world due to its many advantages, including its low density, low cost, good processing ability, and ease of recycling. If PP could be reinforced by proper fillers, its application properties and commercial market would be much larger [1,2,3,4]. The reinforcing effect of fillers on a polymer matrix is closely related to its structure. Fillers such as calcium carbonate, silica, and glass beads improve the modulus of the polymer matrix and cause little damage to the toughness of the material, but they often have a negative influence on the strength [5]. Other fillers with large aspect ratio, such as mica, talc, and wollastonite, can provide a better reinforcing effect by enhancing both the modulus and strength. However, such fillers with large specific surface areas tend to agglomerate and form stress concentration points, leading to decreased toughness. Therefore, the ideal reinforcement material should have a large aspect ratio to meet the requirements of the reinforcement effect, as well as good dispersion and compatibility to avoid forming stress concentration points.

Silicate clay was considered one of the ideal reinforcing fillers in the past. Halloysite naturally possesses a nanotube structure with a high aspect ratio and specific surface area. It is expected to have a good reinforcement effect while maintaining or improving the toughness of the material. Studies have shown that halloysite is an effective reinforcer for many polymers, including polyethylene [6], PP [7], nylon [8], polylactic acid [9], and epoxy resin [10]. Currently, halloysite is still considered a new type of filler for composites. Compared with carbon nanotubes, halloysite is a more commercially viable material for mass-scale industrial applications in the future due to its much lower price and higher production potential [11,12,13]. Wang et al. [14] introduced silicone-filled halloysite into PP composites containing intumescent flame retardants (IFRs) and found that the combination of halloysite and IFR exhibited excellent heat and smoke suppression during the combustion of PP composites. The authors also proposed a crack-bridging effect between halloysite and the PP matrix to explain the elongation improvement brought by halloysite. Wang and Huang [15] investigated the effects of halloysite nanotube orientation on the crystallization and thermal stability of PP nanocomposites. The results showed that both highly oriented and randomly oriented halloysite nanotubes elevated the thermal stability and crystallinity of halloysite/PP composites. Awad [16] reported that the presence of halloysite elevated the crystallinity and crystallization temperature of PP and that a reinforcement effect on mechanical properties can be obtained at a very low content of halloysite (0.5~3 wt.%).

For inorganic minerals, the main disadvantages lie in poor dispersion and interfacial bonding [17]. If the dispersion and interfacial strength could be improved with proper modifications, halloysite would be an excellent nanofiller for many engineering applications. Franciszczak et al. [18] evaluated the effect of halloysite on the mechanical and thermal stability of PP and PP/kenaf fiber biocomposites. Maleic anhydride-grafted PP was employed as a compatibilizer to improve the interfacial bonding and filler dispersion in the composites. The Young’s modulus, flexural modulus, and flexural strength of the composites were enhanced with the introduction of halloysite, while the tensile strength showed no significant change and the Izod impact significantly decreased. Apparent aggregates were observed when the filler loading exceeded 5%. Thus, it is still important and necessary to investigate the proper processing methods to utilize its nanotubular structures in order to obtain a good reinforcement effect with low filling amounts.

In this paper, halloysite was surface-modified by a coupling agent, γ-Aminopropyltriethoxysilane (APTES), through many methods. The influence of silane modification on the morphology, rheology, crystallization behavior, thermal stability, and mechanical properties of halloysite/PP composites was investigated. Finally, the best modification method and optimized filler loading were determined.

## 2. Experimental Section

### 2.1. Materials

Isotactic PP (Pro-Fax 6523, Lyondell Basell, Nanjing, China), with a density of 0.90 g/cm^3^ and a melt flow index of 4.0 g/10 min (ASTM D 1238), was used as the polymer matrix. Halloysite (Al_2_Si_2_O_5_(OH)_4_·nH_2_O) is a tubular structured kaolinite clay mineral with inner and outer diameters of 5–20 nm and 30–50 nm, respectively, and it was provided by Applied Minerals Inc. (Dragonite-HP: KF, Eureka, CA, USA). Chemicals, including toluene and Na_2_CO_3_, were purchased from Aldrich (Shanghai, China) and used as received. APTES was provided by Nanjing Chemical Company (Nanjing, China).

### 2.2. Halloysite Modifications

Halloysite nanotubes were silanized with APTES through both dry and wet modifications. In the dry modification, silane was directly introduced into the composite formulations during the extrusion step, while in the wet modification, halloysite firstly reacted with silane in a dispersion medium, and then the modified halloysite was extracted from the mixture. Usually, proper drying and grinding procedures are needed before the modified halloysite is used as a reinforcing filler.

For wet modification processing, toluene and water were employed as the dispersion media with different routines. In the toluene solution, 45 mL of APTES and 45 g of halloysite clay were added into 750 mL of dry toluene, and the mixture was magnetically stirred for 30 min. The suspension was then refluxed at 120 °C for 20 h for a full reaction. A calcium chloride drying tube was employed to maintain a dry environment in the refluxing system. The modified halloysite was filtered and washed three times with toluene, and it was then finally dried overnight at 120 °C. The modified halloysite here is referred to as TMHA.

Another wet modification method based on a water medium was also adopted in order to further improve the modification efficiency. In total, 30 g of halloysite and 0.9 g of Na_2_CO_3_ were dissolved into 750 mL of distilled water, and the mixture was magnetically stirred at 80 °C for 2 h. A total of 15 mL of APTES was added for the silanization reaction after the pH value of the suspension was adjusted to 8~9 by adding a dilute H_2_SO_4_ solution. The reaction was kept at 80 °C for 4 h. The modified halloysite was filtered and washed three times with distilled water, and it was then finally freeze-dried to a constant weight by a vacuum freeze-drying machine (DC41, Yamoto Scientific, Shanghai, China). The modified halloysite is referred to as WMHA in this study.

### 2.3. Extrusion and Injection Molding

The PP and halloysite were blended by a twin-screw extruder (Haake Polylab System, Thermo Scientific, Waltham, MA, USA). The halloysite was pre-dried at 110 °C for 1 h to remove the surface moisture before extrusion. The temperature profile for the eight heating zones from the hopper to the die was 180/180/185/190/195/200/205/210 °C. The composite strands were cooled through a water bath and pelletized. The pellets were dried for 24 h at 70 °C and then injection-molded in an 80-ton injection press (80 M, BOY Machines, Exton, PA, USA). The injection molding barrel temperature profile from the feed zone to the nozzle was 200/200/200/205/205 °C. The formulations of the composites are listed in Table 1. All the filler contents in this article are based on weight percentage. Considering that halloysite is a nanofiller, it is capable of achieving a significant reinforcement effect at lower addition levels. Proper modification would also improve the interfacial bonding and dispersion of the halloysite to enhance its reinforcement effect. Meanwhile, it has been reported that halloysite agglomeration occurs when the filler loading exceeds 5% [18]. Therefore, a filler concentration of 2% was finally chosen for the following characterizations and measurements.

### 2.4. Characterizations and Measurements

The FTIR spectra of the samples were recorded on a Thermo Nicolet Nexus 670 FTIR spectrometer. In total, 0.1 mg of the sample powder and 50 mg of KBr were mixed, ground, and then pressed into a transparent plate for the measurements. Over 32 scans were collected for each measurement from 400 to 4000 cm^−1^ at a resolution of 4 cm^−1^.

Thermogravimetric analysis (TGA) were performed on a TGA/STAR^e^ system from Mettler Toledo, Switzerland. Approximately 10 mg of the fine ground samples was heated in an aluminia crucible with a heating rate of 5 °C/min under an atmosphere of high purity N_2_.

The microstructure of the halloysite/PP composite was measured by X-ray diffraction (XRD), which was performed on a Scintag X-ray Diffractometer with Cu Kα radiation (*λ* = 1.541 Å, 45 kV, 40 mA) in the 2*θ* range of 5°–35°.

The fracture morphology was observed by a JEOL 6610 Scanning Electron Microscope (Tokyo, Japan) with an accelerating voltage of 10 kV. The specimen was coated with a carbon film to improve its conductivity before observation.

The nonisothermal crystallization of the PP/halloysite composites was measured by differential scanning calorimetry (DSC) on a DSC/STAR^e^ system from Mettler Toledo, Switzerland. All of the DSC measurements were performed under nitrogen purging. The specimens were first heated from room temperature to 210 °C at a heating rate of 10 °C/min, and they were then held at this temperature for 5 min to erase the heat history from previous processes. The specimens were then cooled to room temperature at a rate of 5 °C/min. The heat flows of the specimens during the cooling stage were recorded.

The mechanical properties of the composites were evaluated, including the tensile strength, Young’s modulus, flexural strength, flexural modulus, and Izod impact resistance. The tensile properties were tested according to ASTM D638, specimen type I. The speed of testing was set as 5 mm/min. Flexural properties were tested according to ASTM D790, with the speed of testing of 1 mm/min. Since there was no apparent yield point in the stress–strain curve, the flexural strength was defined as the stress at strain of 7%. The Izod impact resistance was tested according to ASTM D256, method A.

## 3. Results and Discussion

### 3.1. Characterizations of Modified Halloysite and PP-Based Composites

#### 3.1.1. FTIR Analysis

**Figure 1:**
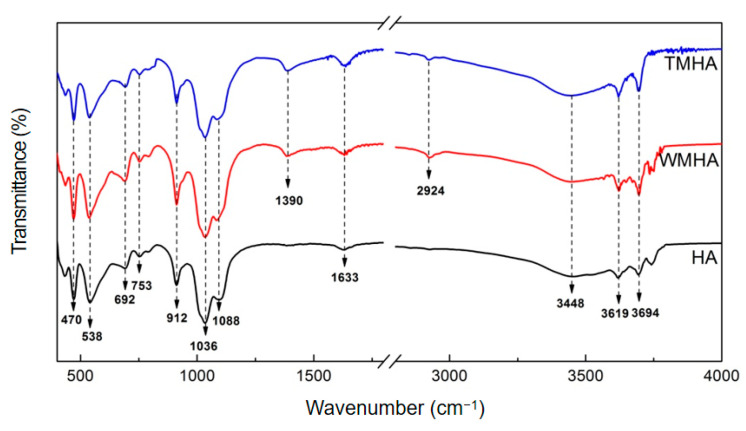
FT-IR spectra of the halloysite and modified halloysite samples.

Figure 1 displays the FTIR spectra of the unmodified and APTES-modified halloysite clay. In Figure 1, the absorption bands at 3619 cm^−1^ and 3694 cm^−1^ correspond to the stretching vibration of the hydroxyl groups of the inner surface of the halloysite. The absorption bands near 3448 cm^−1^ and 1633 cm^−1^ are related to the stretching and bending vibrations, respectively, of the hydroxyl groups from the adsorbed water on the surface of the halloysite. While the peak at 912 cm^−1^ corresponds to the bending vibration of the hydroxyl groups (AlOH), the in-plane stretching vibration band of the Si-O appeared at 1036 cm^−1^ in the silicate layers. Meanwhile, the in-phase stretching band of the apical Si-O bonds appeared near 1088 cm^−1^. The perpendicular vibration bands of the Si-O were located at 753 cm^−1^ and 692 cm^−1^. The absorption bands at 538 cm^−1^ and 470 cm^−1^ were caused by the bending vibration of Al-O-Si and Si-O-Si, respectively [19,20]. After APTES modification, the absorption peak positions and intensities of the Si-O, Al-O-Si, and Si-O-Si in the WMHA and TMHA samples were similar to those of unmodified halloysite, indicating that the silanization modification used in this research did not damage the silicate structure of the halloysite. Meanwhile, compared to unmodified halloysite (HA), the modified clay (WMHA and TMHA) showed some new peaks, which implies the presence of silane. An obvious indicator is the absorption bands in the region of 2924 cm^−1^ and 1390 cm^−1^, which relates to the symmetric stretching and deformation CH_2_ vibration, respectively. It confirmed the surface modification of the halloysite via a silane coupling agent. However, it is also worth noting that the characteristic absorption strength of the water OH stretching (at ~3448 cm^−1^) and the deformation (at ~1633 cm^−1^) remained almost unchanged before or after modification. This demonstrates that only a small part of the OH on the halloysite surface was involved in the silanization reaction.

#### 3.1.2. TGA Analysis

**Figure 2:**
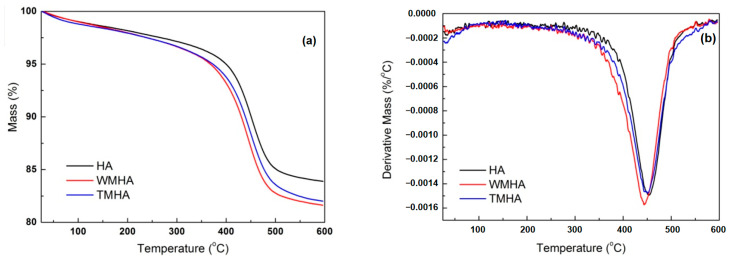
TGA curves of the halloysite before and after silanization: (**a**) mass loss; (**b**) first-order derivative.

The silanization was also proved by the TGA results in Figure 2 and Table 2. As shown in Figure 2a, the influence of the silane modification on weight reduction in the halloysite samples was mainly reflected in the three stages of mass losses from room temperature to 600 °C. The physically adsorbed silane or water on the halloysite surface tends to be released at 50–150 °C, causing larger mass losses in modified halloysite samples (WMHA and TMHA) than unmodified clay (HA). The second mass loss occurring at 150–250 °C relates to the hydrogen-bonded silane molecules, which are more stable than physically adsorbed silane but less stable than grafted silane. From these two mass losses in Table 2, it is clear that only a small part of silane molecules (0.22% for WMHA and 0.11% for TMHA) with such relative weak bonding is left on modified halloysite after washing and drying. Strong bonded silane that has been grafted on the halloysite surface is decomposed at higher temperatures of 250–475 °C, corresponding to the third mass loss. Meanwhile, the dehydroxylation of halloysite mainly occurs between 150–475 °C. Since water molecules mainly exist between the silicate layers of halloysite, the weight loss of dehydration is hardly influenced by surficial silanization. For HA, the weight loss of dehydration is 11.84%. Meanwhile, for WMHA and TMHA, the weight losses at 150–475 °C are 14.43% and 13.20%, respectively, which are significantly higher than those of HA due to the adsorption and grafting of silane. In addition, the total mass losses of halloysite samples reflect the fact that WMHA has a higher grafting ratio of silane than TMHA.

#### 3.1.3. XRD Analysis

**Figure 3:**
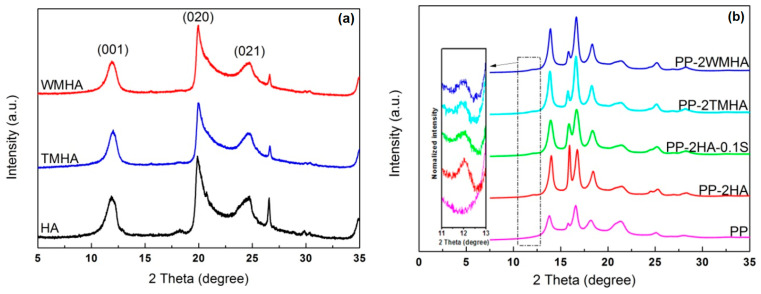
XRD patterns of the halloysite and its composite: (**a**) halloysite; (**b**) halloysite/PP composites.

The XRD patterns of the halloysite are shown in Figure 3a. The basal (001) plane distance of the unmodified halloysite nanotube was approximately 0.72 nm, which corresponds to 2*θ* = 11.9°. This indicates that the halloysite used in this study was completely dehydrated [11]. A dehydrated structure was also confirmed by the presence of reflection peaks at 2*θ* = 19.9° ((020) plane) and 2*θ* = 24.7° ((021) plane) [21]. After silanization processing, the modified halloysite displayed similar diffraction peaks with HA, indicating that halloysite cannot be rehydrated or intercalated by silane molecules. In Figure 3b, diffraction peaks at 2*θ* = 11.9° (corresponding to 0.72 nm of interlayer distance) were also detected in the halloysite/PP nanocomposites, indicating halloysite is not intercalated by PP molecules either. These observations are consistent with results that have already been reported [22].

#### 3.1.4. SEM Observations

**Figure 4:**
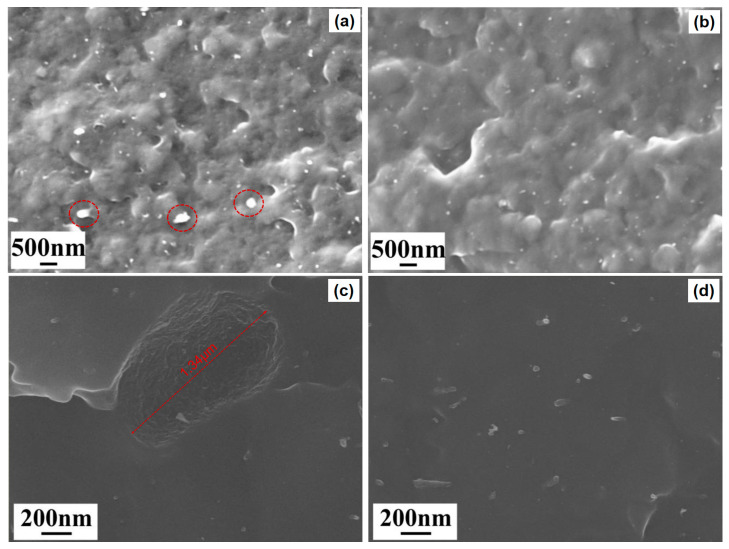
Fracture morphology of the halloysite/PP composites: (**a**) PP-2HA low resolution; (**b**) PP-2WMHA low resolution; (**c**) PP-2HA high resolution; and (**d**) PP-2WMHA high resolution.

The effect of the surface modification of halloysite on the microstructure of the halloysite/PP composites was analyzed by SEM. The fracture morphology of the halloysite-reinforced PP composites is displayed in Figure 4. In Figure 4a, it can be observed that most of the halloysite in the PP-2HA was uniformly dispersed in the PP matrix, but there were still some apparent aggregates (marked in red circles) existing in the samples. It has been reported that such aggregation becomes more severe when halloysite is loaded at higher concentrations [18]. In the high-resolution image shown in Figure 4c, a crater with a rough interface caused by the separation of such aggregates from the PP matrix was also observed, confirming the existence of micro-scale halloysite aggregates in the PP-2HA. Moreover, the rough interface indicates that the halloysite aggregates possess a poor bonding with the matrix. These aggregates tend to be the stress concentrator during deformation. In contrast, the halloysite nanotubes after silanization displayed a better dispersion in the PP-2WMHA. The halloysite was uniformly dispersed in the PP matrix as nanoscale particles, as shown in Figure 4b. In Figure 4d, one can further distinguish the well-dispersed tubular halloysite in the PP-2WMHA. More importantly, the halloysite was found to be well bonded with the PP, indicating that the coupling agent APTES improves the compatibility between inorganic halloysite and organic PP.

### 3.2. Rheology and Crystallization Behaviors

#### 3.2.1. Rheology Behavior

**Figure 5:**
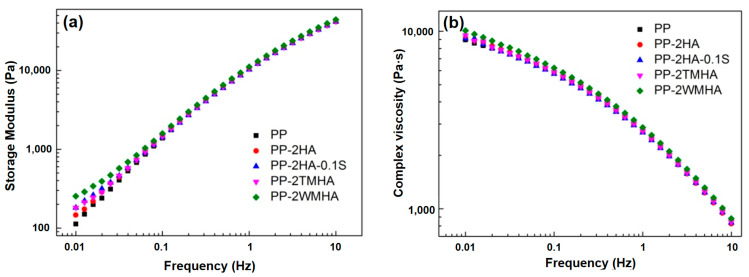
The storage modulus (**a**) and complex viscosity (**b**) of the halloysite/PP nanocomposites.

The storage modulus and complex viscosity of the halloysite/PP composites were measured using an oscillating disc rheometer under the testing mode of the Dynamic Frequency Sweep Test, as shown in Figure 5. It is clear that both the storage modulus and the complex viscosity of the halloysite/PP composites were higher than those of pure PP, especially in the lower-frequency regions (0.01~0.1 Hz), where both properties were more sensitively influenced by the fillers. Halloysite after surface modification displays a better dispersion and interfacial bonding in PP when compared with unmodified halloysite, and it also brings more severe impediments to PP molecular movement in the melt, displaying a relatively higher modulus and viscosity. This is consistent with the results reported in the literature, where the storage modulus and complex viscosity of HA/PP composites do not increase as filler content increases due to the poor dispersion of the halloysite [15].

The storage modulus at low frequencies (at 0.01 Hz, *E’*_0.01_) and its slope in the terminal zone (0.01~0.025 Hz) were obtained from the rheological data in Figure 5a, which are listed in Table 3. The introduction of halloysite into the PP evidently increased *E’*_0.01_ while decreasing the slope, especially in the samples with modified halloysite. It has been reported that higher *E’*_0.01_ and a smaller slope correspond to a better filler dispersion of the nanocomposites [15]. Therefore, the modification methods used in this study are favorable for improving the halloysite dispersion in the PP matrix. Among them, the wet modification methods (PP-2WMHA and PP-2TMHA) exhibited a relatively larger improvement. The results are consistent with the SEM observations.

**Figure 6:**
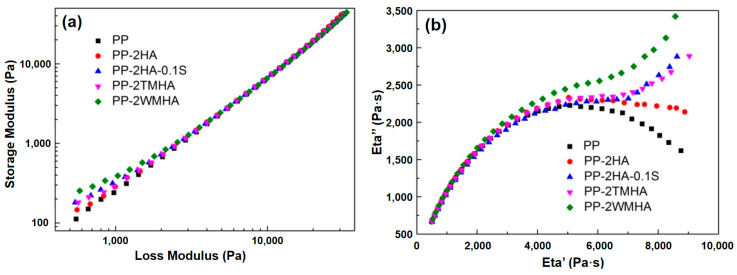
The Han curves (**a**) and Cole–Cole (**b**) curves of the halloysite/PP composites.

The composite microstructure in frequency scanning can be analyzed through the relationship between the storage modulus and loss modulus (the so-called Han curve) [21,23], as shown in Figure 6a. For homopolymers, the curve at low frequencies has been considered to be linear with a slope of 2 [22]. In Figure 6a, it can be observed that the Han curves of the halloysite/PP composites at low frequencies deviated from that of pure PP, which is akin to a straight line. The degree of deviation went in the following ascending order: PP-2HA, PP-2TMHA, PP-2HA-0.1S, and PP-2WMHA. The reason for this phenomenon was that the dispersed halloysite impeded the molecular movements and delayed the molecular relaxation. Such influences were further strengthened when the halloysite was properly modified by a silane coupling agent since silanization improved the interfacial interactions between the halloysite and PP matrix, which plays an important role in molecular movements and relaxation.

The influence of the halloysite on the PP molecular movements was further investigated using the Eta′~Eta″ curve (the so-called Cole–Cole curve, where Eta′ and Eta″, respectively, correspond to the real and imaginary parts of the complex viscosity), which was used to analyze the relaxation behavior of the polymeric materials [24], as shown in Figure 6b. The Cole–Cole curve of the pure PP was approximate to a semicircular arc, which is consistent with the reported results [25]. Meanwhile, for the halloysite/PP nanocomposites, the radius of the arcs increased, indicating that the relaxation was delayed in the composites. For the modified halloysite samples, the Cole–Cole curves at the high-viscosity region (i.e., at low frequencies) warped and displayed increased Eta″. This illustrates that the interfacial bonding was improved by the modification of the halloysite, causing a larger impediment for the PP molecular movements and relaxation after deformation.

Usually, the relaxation behavior can be accurately reflected by the rheological properties of the polymer melt at low frequencies [24]. The relaxation time (*λ*) for pure PP and its composites were calculated with the following equation:

(1) $$\lambda =\frac{{\eta \prime \prime }^{2}}{\eta^{*}G\prime }$$

where *η*″, η^*^, and *G′* denote the imaginary part of the complex viscosity, the complex viscosity, and the storage modulus of the tested materials at a frequency of 0.01 Hz, respectively. The calculated relaxation time for PP was 2.6 s. Meanwhile, for PP-2HA, PP-2HA-0.1S, PP-2TMHA, and PP-2WMHA, the relaxation time was 3.4 s, 5.0 s, 4.8 s, and 6.4 s, respectively. These values were apparently larger than that for PP, which is consistent with the trend of the Cole–Cole curves in the high-viscosity region in Figure 6b.

#### 3.2.2. Nonisothermal Crystallization Behavior

**Figure 7:**
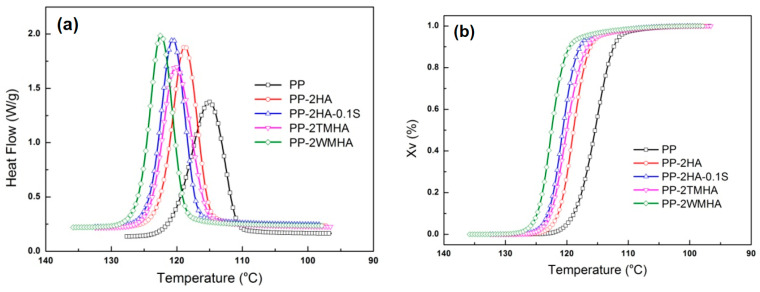
The nonisothermal crystallization of the halloysite/PP nanocomposites: (**a**) heat flow; (**b**) relative crystallinity.

Figure 7 presents DSC thermograms of the halloysite/PP nanocomposites during the cooling cycle from the melt state at a rate of 5 °C/min. The crystallization onset temperature (*T_co_*), peak temperature (*T_cp_*), crystallization enthalpy (Δ*H*), crystallinity (*X_c_*), half-crystallization time (*t*_0.5_), and corresponding temperatures (*T*_0.5_) were calculated and are summarized in Table 4. It can be seen that the addition of halloysite promoted the crystallization of the PP by showing higher *T_co_* and *T_cp_* in the HA/PP composites. This phenomenon is mainly caused by the heterogeneous nucleation effect of halloysite filler. Hence, the crystallization of the halloysite/PP composites was accomplished at relatively higher temperature ranges. It further led to higher Δ*H* and *X_c_* in the halloysite/PP composites since higher temperatures facilitate the molecules’ diffusion and mobility to form perfect spherulites. The promotion effect of halloysite on crystallization has also been reported in the literature [15,16]. Meanwhile, the spherulite growth rate after nucleation was only affected by the temperature, and it decreased with increasing temperature. Thus, the value of *t*_0.5_ was not a direct parameter for evaluating the crystallization rate of the Neat PP and its composite at different temperatures. *T*_0.5_, defined as the crystallization temperature corresponding to 50% crystallinity, was used to reflect the influences of the halloysite and its modification on the crystallization rate. Evidently, the addition of halloysite promoted the crystallization rate of the PP, showing higher *T*_0.5_. Such a promotion was further enhanced by silane modification of the halloysite. The reason for this may lie in the better dispersion and chemical bonding of the WMHA and TMHA, which provide denser and more stable nucleation centers for the PP matrix, in the composites.

The relative crystallinity of PP and its composites is illustrated in Figure 7b. Avrami crystallization theory was used to analyze the kinetic data, which are summarized in Table 4. It was observed that Avrami *t*_0.5_ had good agreement with the experimental results. PP-2HA, which may possess a balanced nucleation effect and spherulite growth rate, had the smallest *t*_0.5_. The change in the overall crystallization rate *K_c_* demonstrated the temperature effect on the spherulite growth rate. The nuclei in the composites formed at higher temperatures grew more slowly and, ultimately, resulted in smaller *K_c_* values. The Avrami exponent *n*, which contains information on nucleation and growth geometry, was larger than its theoretical values. Similar results were reported by other studies when they investigated the non-isothermal crystallization behavior of polymers [26]. The reason for this may be the fast rate of the primary crystallization in halloysite composites and the difference in the physical meaning of *n*, which is derived from isothermal crystallization.

### 3.3. Thermal and Mechanical Properties

#### 3.3.1. Thermal Stability

The thermal stability of the halloysite/PP composites was investigated by TGA. The temperatures corresponding to mass losses at 5% (*T*_5%_), 10% (*T*_10%_), and the maximum rate (*T*_max_) during the decomposition are summarized in Table 5. It was evident that *T*_5%_, *T*_10%_, and *T*_max_ were elevated with the introduction of halloysite (PP-2HA). Meanwhile, *T*_5%_ and *T*_max_ were further improved after the halloysite was modified with silane. The main contribution of the halloysite to the thermal stability came from the dehydration during the decomposition. The corresponding mass loss of water could be up to 15%~20%, thus preventing flames and retarding combustion [27]. In addition, the tubular structure of halloysite could trap the small molecules produced from the polymer decomposition, postponing the mass transportation. It becomes the dominating mechanism when improving the thermal stability at high temperatures when the dehydroxylation is almost completed. Such a trap mechanism is influenced by the dispersion of halloysite nanotubes. Apparently, the better the halloysite dispersion, the better the thermal stability brought by the tubular traps. Compared with the literature, the wet modifications used in this study improve the dispersion of halloysite nanotubes and facilitate the tubular trap, yielding enhanced thermal stability [15].

#### 3.3.2. Mechanical Properties

**Figure 8:**
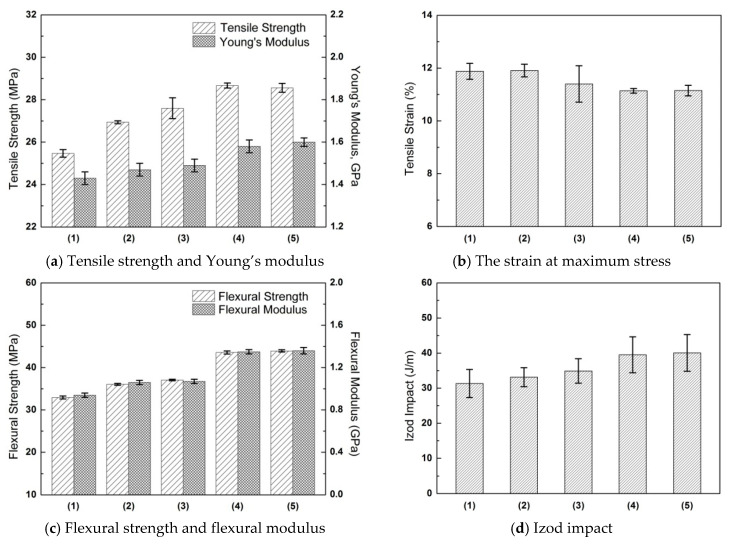
The mechanical properties of the PP and halloysite/PP composites: (1) PP; (2) PP-2HA; (3) PP-2HA-0.1S; (4) PP-2TMHA; and (5) PP-2WMHA.

Figure 8 exhibits the mechanical properties of the halloysite/PP composites, including the tensile strength, Young’s modulus, the strain at maximum stress, flexural strength, flexural modulus, and the notched Izod impact resistance. The introduction of unmodified halloysite improved the tensile strength, flexural strength, and flexural moduli. The strengths and moduli were further improved after the dry modification and wet modification. It has been proven that wet modification facilitates the dispersion and interfacial bonding of halloysite, thus displaying a better reinforcement effect. Moreover, the halloysite tends to promote the heterogeneous nucleation of PP, facilitating the formation of a fine spherulite structure and increasing the crystallinity of PP. These influences are amplified by wet modification, which further enhances the strength and modulus of the composites. The reinforcement effect of the halloysite was also reported by other studies [14,16,18]. The APTES modification used in this study can achieve more significant reinforcement effects on the tensile strength, Young’s modulus, and flexural strength when compared with directly adding the compatibilizer (maleic anhydride-grafted PP) [16].

It is also worth noting that the notched Izod impact resistance was also improved with the introduction of halloysite, and it also displayed a similar trend when the halloysite was modified with a silane coupling agent. This was quite different to the traditional fillers like calcium carbonate, talc, and glass beads. The reason could be attributed to the good interfacial bonding, good dispersion, high crystallinity, and fine spherulite structure. The literature has proposed that the formation of crack bridging between halloysite and the matrix can increase the toughness of halloysite/PP composites [14]. Such crack bridging can be enhanced by improved interface bonding and filler dispersion, for which the APTES modification that was used in this study has been proven to be effective.

**Figure 9:**
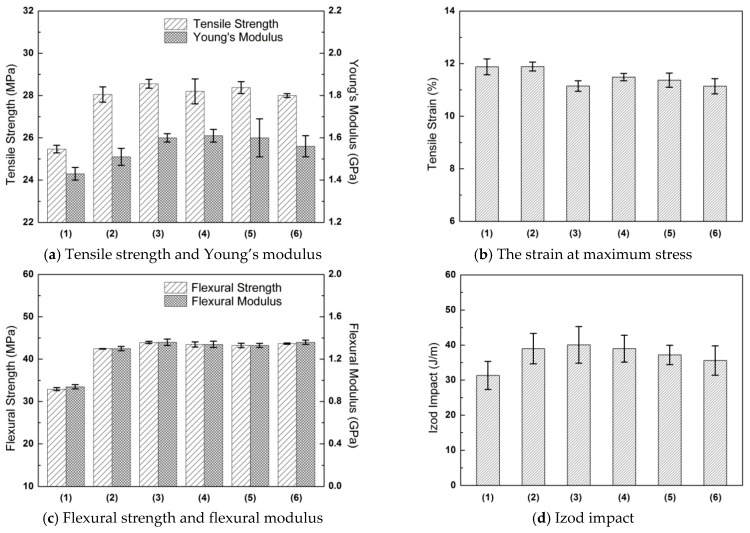
The mechanical properties of the halloysite/PP composites with different loading: (1) PP; (2) PP-1WMHA; (3) PP-2WMHA; (4) PP-3WMHA; (5) PP-4WMHA; and (6) PP-5WMHA.

Samples using wet modifications (PP-2WMHA and PP-2TMHA) demonstrated similar influences on the reinforcement of halloysite. Considering the fact that modification for WMHA has a much higher production efficiency, the method was more beneficial for engineering applications. Therefore, the WMHA content was also optimized according to mechanical properties shown in Figure 9. It could be observed that 1% halloysite could bring evident improvement to the strength and modulus of PP composites. The mechanical properties reached peak values as the halloysite content increased to 2%. Higher halloysite loading did not bring further reinforcement for PP. With a halloysite loading of 5%, the tensile strength and impact resistance became even worse. High loading levels are not beneficial for balanced mechanical properties since nanofiller tends to form aggregates that ultimately deteriorate the mechanical properties. The form of aggregates has been also reported in the literature to be harmful to the reinforcement effect [18]. Therefore, PP reinforced with 2% of modified halloysite WMHA possesses balanced mechanical properties. The tensile strength, Young’s modulus, flexural strength, and Izod impact resistance were improved by 13%, 12%, 33%, and 28%, respectively, with the reinforcement of 2% WMHA when compared to Neat PP.

## 4. Conclusions

In this study, halloysite was successfully grafted with the coupling agent APTES using both wet and dry modification methods. The rheological properties, crystallization behavior, thermal stability, and mechanical properties were investigated to evaluate the influence of halloysite modification on the reinforcement of halloysite/PP nanocomposites. The following main conclusions can be drawn.

1. Silane was successfully grafted onto the halloysite surface using both dry and wet modification methods. Among them, wet modification using water solution displayed the higher grafting ratio. SEM observations demonstrated that silanization improved the dispersion and interfacial bonding between the halloysite and PP matrix.
2. After modification, the low-frequency storage modulus of the halloysite/PP composites increased and the slope decreased, indicating that silane modification improved the dispersion of the halloysite in the matrix. The modification also enhanced the interfacial interactions between the halloysite and PP by showing an apparently prolonged relaxation time of the composites. Meanwhile, the crystallization behavior and thermal properties demonstrated that the modification also elevated the crystallinity and thermal stability of the halloysite/PP composites.
3. Both wet and dry modifications improved the mechanical properties of the halloysite/PP nanocomposites, with wet modification exhibiting a stronger reinforcement effect than dry modification. WMHA was further optimized by the addition of fillers in the nanocomposites, and it was found that the optimal mechanical performance was achieved with a loading of 2%.

## Figures and Tables

**Table 1:** The formulations of the composites.

Samples	Filler and Modification	Filler Content, wt.%
Neat PP	No filler	0
PP-2HA	Unmodified	2
PP-2HA-0.1S	Dry modification	2
PP-2TMHA	Wet modification/toluene	2
PP-2WMHA	Wet modification/water	2

**Table 2:** Mass loss data for the halloysite before and after silanization.

Mass Losses, %	HA	WMHA	TMHA
50~150 °C, %	1.00	1.13	1.07
150~250 °C, %	0.92	1.14	1.03
250~475 °C, %	10.92	13.29	12.17
Total Mass Loss, %	12.84	15.56	14.27

**Table 3:** The *E’*_0.01_ and slope in the terminal zone of the halloysite/PP nanocomposites.

Samples	*E’*_0.01_, Pa	Slope
PP	112	1.09
PP-2HA	146	1.03
PP-2HA-0.1S	181	0.79
PP-2TMHA	181	0.74
PP-2WMHA	253	0.67

**Table 4:** The nonisothermal crystallization kinetic data of the halloysite/PP nanocomposites.

Samples	*T_co_*	*T_cf_*	*T_cp_*	Δ*H*	*X_c_*	*t*_0.5_	*T*_0.5_	Avrami Parameters			
	°C	°C	°C	J/g	%	s	°C	*n*	*K_c_*	R^2^	*t*_0.5_
PP	127.6	96.5	115.1	92.1	44.1	150	115.4	5.4	0.34	0.9999	151
PP-2HA	130.2	97.2	118.7	97.0	46.4	141	118.9	6.7	0.29	0.9998	142
PP-2HA-0.1S	132.5	98.3	120.6	97.7	46.7	147	120.6	7.6	0.24	0.9998	147
PP-2TMHA	132.4	96.4	120.2	97.4	46.6	155	120.0	7.6	0.22	0.9999	154
PP-2WMHA	135.8	97.6	122.4	98.1	47.0	166	122.4	8.7	0.16	0.9997	166

**Table 5:** The thermal stability of the halloysite/PP composites.

Samples	*T*_5%_, °C	*T*_10%_, °C	*T*_max_, °C
Neat PP	403.5	419.0	453.7
PP-2HA	403.1	425.2	456.3
PP-2HA-0.1S	404.7	422.6	461
PP-2TMHA	404.1	423.5	464.5
PP-2WMHA	407.4	424.7	463.9

## Data Availability

Data will be made available upon request.
